# 

**Published:** 1913-04-15

**Authors:** 


### ANNOUNCEMENTS.

Dental Society Meetings in May: New York State, at Albany, May 8th to 10th.

Massachusetts State at Boston, May 8th to 10th.

Illinois State at Peoria, May 13th to 16th.

North Dakota State at Fargo, May 13th and 14th.

Texas State at Temple, May 15th to 17th.

Nebraska State at Omaha, May 19th to 22nd.

The Susquehanna Dental Association meets at Wilkes-barre, Pa., May 20th to 22nd.

Indiana State at Indianapolis, May 20th to 22nd.

Vermont State at Burlington, May 21st to 23rd.

Kentucky State at Lexington, May 26th to 28th.

Southern Branch of the National Dental Association. The 15th annual meeting of the Southern Branch of the National Dental Association will be held at the Chamberlin Hotel, Old Point Comfort, Va., July 22nd to 25th, inclusive. The Virginia State Dental Society will meet conjointly with the Southern Branch at that time.

Thos. T. Moore, Jr.,
*Cor. Sec. So. Br., of N. D. A.*

Alumni Association of the Washington University Dental School. The Alumni Association of the Washington University Dental School (Missouri Dental College), St. Louis, Mo., has decided to withdraw the annual clinic for 1913 owing to the proximate date of the next session of the National Dental Association at Kansas City, Mo.

R. H. Miller, D.D.S.,
Chairman Publicity Committee.

NORTHERN OHIO MEETING. The fifty-sixth annual meeting of the Northern Ohio Dental Association will be held at Cleveland, June 5, 6 and 7.

C. D. PECK, D.D.S., *Sec'y.*

NATIONAL DENTAL ASSOCIATION. The 1913 session of the National Dental Association will be held in Kansas City, Mo., July 8th to 11th. The Local Committee of Arrangements have selected the Baltimore Hotel as "Headquarters" and made the other necessary arrangements for this meeting. The officers and committees are planning to present an exceptionally interesting program, the details of which, together with the other arrangements, will be present in later Journals.

FRANK O. HETRICK, *President,*
Ottawa, Kansas.
HOMER C. BROWN, *Rec. Sec'y,*
185 East State Street,
Columbus, Ohio.

NEW EDITOR OF WESTERN DENTAL JOURNAL. Dr. Charles C. Allen, Secretary of the Kansas City Dental College takes the place of the late Dr. Joseph Root as editor of this Journal. Dr. Allen is a writer of considerable experience and an excellent executive. He should make good as an editor, and we cordially welcome him to the ranks and wish him all success possible.

DR. F. X. BUXBAUM, a New York dentist is suing the Guggenheim estate for services to the daughter in setting a splint for a fractured jaw, in the sum of $7500.00. Dr. Buxbaum formerly practiced in Cincinnati.

